# Fast optical method for characterizing plasmonic nanoparticle adhesion on functionalized surfaces

**DOI:** 10.1007/s00216-019-02307-x

**Published:** 2019-12-24

**Authors:** László Mérai, László Janovák, Dániel Sándor Kovács, Imre Szenti, Lívia Vásárhelyi, Ákos Kukovecz, Imre Dékány, Zoltán Kónya, Dániel Sebők

**Affiliations:** 1 Interdisciplinary Excellence Centre, Department of Physical Chemistry and Materials Science, University of Szeged, Rerrich Bela sqr 1, Szeged, 6720 Hungary; 2 Interdisciplinary Excellence Centre, Department of Applied and Environmental Chemistry, University of Szeged, Rerrich Bela sqr 1, Szeged, 6720 Hungary

**Keywords:** Plasmonic nanoparticles, Adhesion, Optical method, Flow system, Surface charge, Reflectometric interference spectroscopy

## Abstract

In this paper, a rapid optical method for characterizing plasmonic (gold) nanoparticle (AuNP) adhesion is presented. Two different methods were used for AuNP preparation: the well-known Turkevich method resulted in particles with negative surface charge; for preparing AuNPs with positive surface charge, stainless steel was used as reducing agent. The solid surface for adhesion was provided by a column packed with pristine or surface-modified glass beads. The size of the nanoparticles was studied by transmission electron microscopy (TEM) and small-angle X-ray scattering (SAXS); the surface charge of the components was determined by streaming potential measurements. The characterization of adhesion was performed in a flow system by UV-Vis spectroscopy. During the adhesion experiments, the role of the surface charge, the particle size, and the pH were studied, as well as the adhered amount of gold nanoparticles and the surface coverage values. The latter was estimated by theoretical calculations and defined by the quotient of the measured and the maximal adhered amount of nanoparticles, which could be determined by the cross-sectional area of the NPs and the specific surface area of the glass beads. The results are verified by the polarization reflectometric interference spectroscopy (PRIfS) method: silica nanoparticles with diameters of a few hundred (*d*~450) nanometers were immobilized on the surface of glass substrate by the Langmuir–Blodgett method, the surface was modified similar to the 3D (continuous flow packed column) system, and gold nanoparticles from different pH solutions were adhered during the measurements. These kinds of modified surfaces allow the investigation of biomolecule adsorption in the same reflectometric setup.

**Electronic supplementary material:**

The online version of this article (10.1007/s00216-019-02307-x) contains supplementary material, which is available to authorized users.

**Figure:**
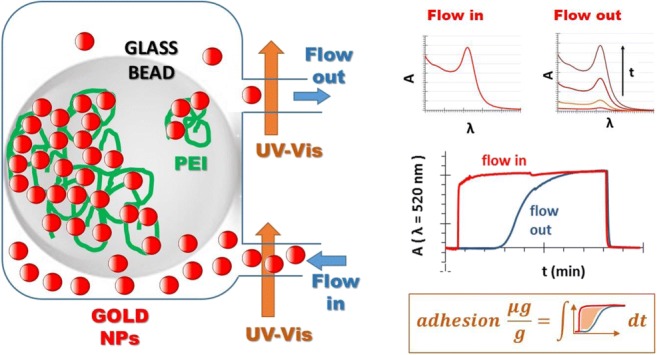
Graphical abstract

## Introduction

Plasmonic particles are widely investigated due to their optical trapping capabilities. In the last few decades, several fields of application arose, including sensitized solar cells [1], optical circuits [2], and analytical methods, such as surface-enhanced raman spectroscopy and surface plasmon resonance spectroscopy [3, 4]. Visible-light photocatalysis also deserves a mention: the presence of Au or Ag nanoparticles can tune the bandgap energy of semiconductors like anatase TiO_2_, which is originally excitable by near-UV electromagnetic radiation [5–7].

As the popularity of this field grew, many simple and scalable methods have been published discussing the preparation of plasmonic nanostructures, including, for example, bottom-up solvent-phase syntheses [8, 9] and top-down lithographic methods [10], which both offer good control over morphological properties and functionalization. While there are many plasmonic materials to apply including Al, Pt, or Ag, the use of Au dominates these fields [11].

In most of their sensing applications, plasmonic metal nanoparticles require an adhesion layer made of another metal such as Cr or Ti to promote their binding to common substrates like glass [12]. The adhesion can be improved for example by thermal annealing [13] or binding the nanoparticles to the surface covalently [14]. Although the adhesive forces between the plasmonic particles and glass are generally weak, porous glass substrates with high specific surface area were proven to be effective in sensing capability enhancement [15]. If the adhesion takes place at a solid/liquid (S/L) interface, one must consider the effect of surface charges of both the particles and the substrate and the particle-particle interactions. Kállay and his co-workers extensively studied the pH- and functionalization-dependent adhesion strength, kinetics, and stability of colloidal and nanoparticles in continuous-flow packed column systems. Using continuously rinsed packed columns, they provided information on the interaction energy profiles and diffusion characteristics of their model systems, studying the particle attachment and detachment kinetics [16–20]. As the concentration of plasmonic Au nanoparticles is easily traceable and the stability of their dispersions is strongly dependent on their chemical environment (pH, ionic strength, complexing agents, etc.) [21], they could be excellent test subjects in such model systems.

In this paper, we present a methodology to characterize pH-, shape-, and functionalization-dependent adhesion strength of model AuNPs as plasmonic nanoparticles, and theoretically any other plasmonic metal and even metal-oxide nanoparticles in simple continuous flow systems. As the modern enhanced oil recovery processes (EOR) utilize metal-oxide nanoparticles beside surfactants and polymers to further increase the oil recovery rate [22, 23], the presented methodology could potentially be helpful during the optimization of novel EOR processes. Furthermore, in this work also the polarization reflectometric interference spectroscopy (polarization RIfS, PRIfS) method [24] is used to verify the results. The concept of the PRIfS technique is similar to the well-known label-free optical analytical methods, such as RIfS [25–36] and surface plasmon resonance (SPR) [37–40]. To the best of our knowledge, this is the first report of applying PRIfS technique for the characterization of nanoparticle adhesion in an aqueous flow system.

## Experimental

### Materials

#### Preparation of gold nanoparticles

Negatively surface charged gold nanoparticles [AuNP(−)] with an average diameter of 14 nm were synthesized by the well-known Turkevich method [41]. Positively surface charged AuNP(+) with an average diameter of 51 nm were synthesized by the method presented in [42]. The pH of the AuNP(−) gold sol (pH = 5) was adjusted by adding *c* = 0.1 M HCl (for pH = 3) or *c* = 0.1 M NaOH (for pH = 7 and 9) solutions to the original sol.

#### Preparation of the SiO_2_ particles

Monodisperse *d*_av_ = 450-nm silica particles were prepared by the modified method of Jezequel et al. according to [43].

#### Surface functionalization of the glass beads

Glass beads (< 106 μm and 212–300 μm, average diameters ~ 50 and ~ 250 μm, respectively, acid-washed, Sigma–Aldrich; density 2.52 g/cm^3^) were used as a solid surface for gold nanoparticle adhesion experiments. Cationic polyethylenimine (PEI, branched, MW~8000, Aldrich) was used as a positively charged polyelectrolyte for functionalization (and charge alteration) of the glass beads.

### Instrumental methods

#### Transmission electron microscopy

TEM measurements were carried out by a FEI TECNAI G2 20 X-Twin high-resolution transmission electron microscope (equipped with electron diffraction apparatus) operating at an accelerating voltage of 200 kV.

#### Small-angle X-ray scattering

The small-angle X-ray scattering (SAXS) curve was recorded with a slit-collimated Kratky compact small-angle system (KCEC/3 Anton-Paar KG, Graz, Austria) equipped with a position-sensitive detector (PSD 50 M from M. Braun AG Munich, Germany) containing 1024 channels 55 μm in width. CuKα radiation (λCu_Kα_ = 0.1542 nm) was generated by a Philips PW1830 X-ray generator operating at 40 kV and 30 mA.

#### Computed tomography

Micro-CT measurements were carried out with a Bruker SkyScan 2211 nanoTomograph instrument (source voltage, *U* = 110 kV; source current, *I* = 150 μA; pixel resolution 1.6 μm). A capillary (Markröhrchen, Hilgenberg) 2 mm in diameter was filled with the *d*_av_ = 250 μm glass beads. After the reconstruction procedure (NRecon software, Bruker), CTVox and CTAn (Bruker) softwares were used to generate the 3D-rendered image and calculate the size distribution and porosity (*ε*).

#### Streaming potential

The streaming potential values of the stationary phase (glass beads), the AuNP(−)s and AuNP(+)s were measured applying a PCD-04 Particle Charge Detector (Mütek Analytic GmbH, Germany). The negative surface charge excess of the glass beads and the AuNP(−)s were neutralized by 0.01 g/100 cm^3^ (0.01%) polyethylene imine (PEI) and 0.01% hexadecylpyridinium chloride (HDPCl) solutions, respectively, while the positive surface charge excess of AuNP(+)s was neutralized by 0.01% sodium dodecylsulfate (NaDS) surfactant solution during particle charge titrations. The charge compensation states were achieved at streaming potentials of 0 mV. The surface charge excess values were calculated according to the following equation:

1 $$ {q}_1=\frac{V_20,01{c}_2{q}_2}{c_1{V}_1{M}_1} $$

where q_1_ and q_2_ are the specific charge excess of the examined specimen and the titrant, respectively (meq/g) *V*_2_ and *c*_2_ are the volume (cm^3^) and the concentration (g/100 cm^3^) of the charge-neutralizing agent solution, *V*_1_ and *c*_1_ are the volume (cm^3^) and the concentration (mol/dm^3^) of the examined dispersion, while *M*_1_ is the molar mass (g/mol) of the examined specimen. The reversal of surface charge excess is also available by adding an excess amount of titrant to the initial dispersion.

#### Spectrophotometry

The visible range extinction spectra of the gold sols were recorded by an ADC1000-USB diode array spectrophotometer (Ocean Optics, Duiven, Netherlands). The data points of the extinction vs. concentration calibration curves are the average of five repeated measurements, the uncertainty of the measured extinction values is ± 0.025.

#### Adhesion measurement

**Fig. 1:**
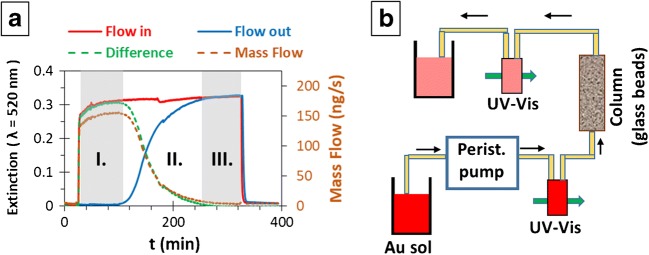
The principle of the gold nanoparticle adhesion measurement (**a**) and the schematic view of the measurement setup (**b**)

Figure 1 shows the principle (a) and the schematic view of the adhesion measurement setup (b). The flow of the gold sol (*c* = 20 mg/L) is driven by a peristaltic pump (Ismatech Reglo Digital) with a flow rate of *I* = 500 μL/min through a stationary phase (*L* = 60 mm long and *d* = 8 mm in diameter glass tube filled with glass beads, *V* = 3.016 mL), while the extinction of the gold sol is measured both before (flow-in) and after (flow-out) the column with *V* = 400 μL flow-through cells. The difference between the flow-in and flow-out concentrations is due to the adhesion. In dilute systems the extinction of the sol is proportional to the concentration of the plasmonic particles; therefore, the former can be converted to the latter by calibration (see Electronic Supplementary Material (ESM) Fig. S1). It has to be noted that the uncertainty of ± 0.025 in the extinction value causes uncertainties of 1.47 and 3.26 mg/L in the concentration of AuNP(−) and AuNP(+) sols, respectively, during the adhesion measurements, which are indicated by the X- and Y-error bars on Fig. S1 (see ESM). During the measurement the extinction values corresponding to the characteristic plasmonic peaks are monitored in real time, the signal/noise ratio is improved by polynomial fitting, as described in [31]. The point-by-point substraction of the flow-out curve from the flow-in curve results in the difference curve (see Fig. 1). The (extinction-) difference (E) curve can be converted to mass flow (MF) by eq. (2):

2 $$ MF(t)=c(t)\cdotp I=E(t)\cdotp k(c)\cdotp I $$

where *MF* is the mass flow, *c* is the concentration and *E* is the extinction in a given *t* moment, *k(c)* is the slope of the *c* vs. *E* calibration curve (see ESM Fig. S1) and *I* is the constant flow rate (0.5 mL/min). This curve can be divided into three characteristic ranges: (1) in the first range (I. on Fig. 1) the mass flow through the flow-out cell is zero, i.e., all of the gold nanoparticles are adhered to the GB surface, the extent and rate of the adhesion process is maximal; (2) in the second regime (II. on Fig. 1) the adhesion rate decreases, more and more gold nanoparticles are passing through the stationary phase; (3) the third region (III. on Fig. 1) is the saturation range, the adhered amount of AuNPs reached the maximal adhesion capacity of the GBs, all of the particles pass through the column. The adhered amount of gold nanoparticles (*m*^*NP*^_*exp*_) can be obtained by the numerical integration of this mass flow curve. It has to be noted that there are two sources of inaccuracy in the flow system: on one hand, there is a path, therefore a time difference between the two flow-through cells, which was *t* = 170 ± 5 s depending on the space filling of the column (1-ε). The flow-out curves were corrected by this delay. On the other hand, the saturation of the concentration profile in the flow-through cells is not an instantaneous process; it takes *t* = 50 s, which causes ca. m^NP^_exp_ = 10 μg AuNP/g GB deviation. This inaccuracy is indicated in the tables.

#### Thin film preparation

Langmuir films at the air/liquid interface and LB films on glass substrates (for the reflectometric interference spectroscopy measurements) were prepared in a Kibron MicroTroughS Langmuir-trough. Spreading sols were obtained by mixing the original sol with chloroform (Sigma–Aldrich, Chromasolv®, ≥ 99.9%) in the volume ratio 1:1. Glass substrates for LB films were cleaned in piranha solution and rinsed with deionized water before film preparation. The monolayer films were pulled out at constant pressure (*π* = 10 mN/m), only in the upstroke direction. The surface of the thin films was modified (with PEI) similarly to the 3D system.

#### PRIfS

Polarization reflectometric interference spectroscopy measurements were carried out according to the phenomenon and optical setup described in [24]. RIfS and PRIfS methods are hardly different, the only difference lies in the measurement techniques. While in the former case the detector measures the average of all the polarization states, the ratio of the s- and p-polarized reflected intensity is measured by using a polarizer in the latter. Therefore, the sensor response (*R*) is defined by the ratio of the p- and s-polarized components of the reflected light, as described in eq. (3):

3 $$ R=\frac{I_{R,p}}{I_{R,s}} $$

## Results and discussion

### Characterization of the gold nanoparticles

**Fig. 2:**
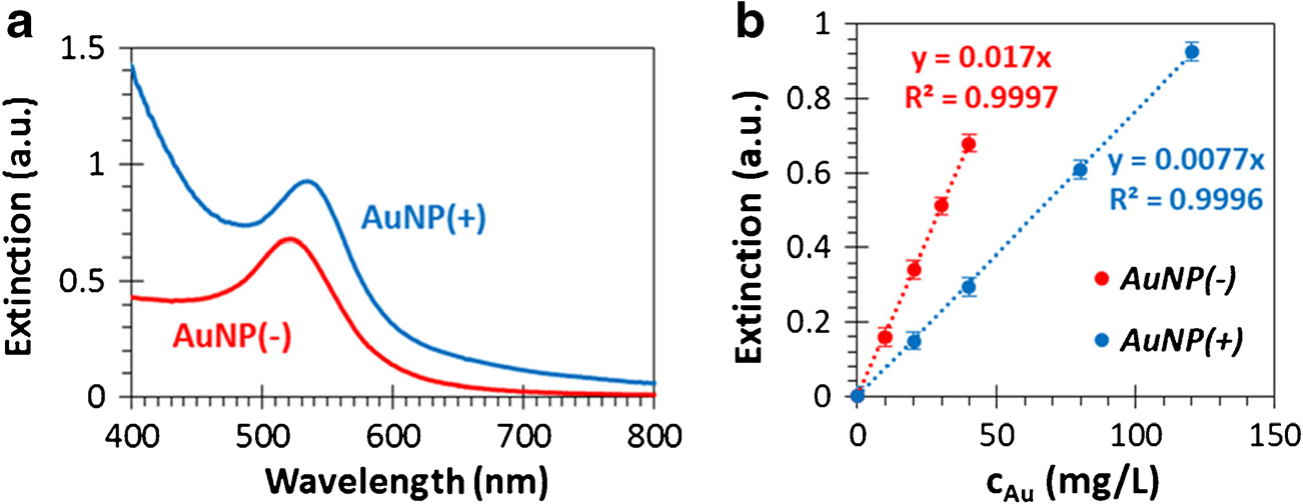
Representative UV-Vis spectra of the AuNP(−) (red) and AuNP(+) (blue) sols (**a**) and the extinction vs. concentration (calibration) curves at *λ* = 520 (red) and 532 nm (blue) for AuNP(−) and AuNP(+) sols, respectively

Figure 2a shows the representative UV-Vis extinction spectra of the synthesized gold nanoparticles/sols. There is a characteristic plasmonic band at *λ* = 490–550 nm (with a maximum at *λ* = 520 nm) in the case of the monodisperse AuNP(−) sol with narrow size distribution. The plasmonic band appears at *λ* = 505–565 nm (with a maximum at *λ* = 532 nm) for the AuNP(+) sol, which corresponds to the formation of larger particles compared with the AuNP(−) sol. During the adhesion measurements in the flow system the extinction values corresponding to these wavelengths (*λ* = 520 and 532 nm for AuNP− and AuNP+, respectively) are monitored, because the calibration curves (i.e., extinction vs concentration, see Fig. 2b) were measured and plotted on these same wavelength values.

The size of the AuNP(−) and AuNP(+) plasmonic gold nanoparticles were determined by TEM measurements (ESM Fig. S2A-D), moreover, in the case of the AuNP(−) sample, due to its monodispersity, the results could have been confirmed by SAXS measurements (ESM Fig. S2.E). On the TEM image and size distribution diagram the nearly monodisperse character of the AuNP(−) sample with an average diameter of *d* = 14 ± 1 nm can be observed, however, the positively charged gold particles have a broad size distribution between 30 and 80 nm with an average of ~ 51 ± 13 nm. Furthermore, the Guinier plot [44] of the AuNP(−) sample’s SAXS curve shows the monodispersity with a diameter of *d* = 14.7 nm; the slight difference can be explained by the citrate as a stabilizing agent.

### Characterization of the glass beads

Determining the size distribution of the used glass beads contributes to the accurate estimation of the specific surface area, the surface coverage (Θ) of the AuNPs and space filling of the stationary phase. Size distributions of the smaller (~ 50 μm) and larger (~ 250 μm) glass beads were determined by optical microscopy (ESM Fig. S3.a). The average diameter of the smaller fraction is 55.7 ± 9.2 μm and it is 273.7 ± 37.6 μm for the larger glass beads (ESM Fig. S3.b). Therefore, for practical reasons the notations of the used glass beads will be GB56 and GB274, respectively. Similarly, the notations of the surface-modified samples are GB56(+) and GB274(+), respectively. The size distribution of the GB274 glass beads was verified by micro-CT measurements: ESM Fig. S3c shows the 3D-rendered volume of the capillary filled by GB274 spheres, while ESM Fig. S3d represents the selected volume of interest (VOI) for further calculations (red: GB274, blue: void). Based on μCT measurements the size distribution of the larger fraction was determined (which hardly differs from the optical microscopy results), as well as, the average porosity of the stationary phase was calculated (ε = 36.8%, ESM Fig. S3e). This data is consistent with the values presented later, namely the porosity of the different stationary phases determined by mass measurement.

### Streaming potential measurements

The specific surface charge excess value of the AuNP(−) particles was calculated according to charge compensation method described in Chapt. 2.2. The charge compensation required 295 μL of 0.01% HDPCl solution, which according to our equation gives a specific surface charge excess value of −4.18 **×** 10^−2^ meq/g, while the value of the independently determined ζ-potential is −43.2 mV. In the case of AuNP(+) particles, 0.01% NaDS solution was used during the surface charge measurements: the volume of the solution required for charge neutralization turned out to be 9.5 mL, indicating a specific surface charge value of 1.64 eq/g, while the ζ-potential was 47.6 mV.

The surface charge excess of the GB56 glass beads, used as adhesion surfaces was also reversed applying excess amount of PEI (0.01 wt. PEI solution). In this case, the consumed 350 μL of 0.01% HDPCl solution during charge compensation measurements indicated the specific surface charge value of −1.96 **×** 10^−5^ meq/g. However, this charge neutralization method cannot be applied on GB274 glass beads because of the fast sedimentation of the beads.Therefore, we assumed lower specific surface charge excess values (for geometric reasons, assuming that the same amount (mass) of the larger GBs has a smaller total surface area), keeping the same amount of used PEI as in the case of the smaller GB56 glass beads during the surface charge reversal process.

### Theoretical calculations

Theoretical calculations were performed to estimate the specific surface area (A^s^_φ_, m^2^/g) of the glass beads and the cross-sectional surface area (a^s^_NP_, m^2^/g) of the gold nanoparticles. The ratio of these two values is in correlation with the maximal (monoparticular) surface coverage (Θ) of the AuNP/GB system. The specific surface area of a spherical particle depending on the particle diameter can be determined by eq. (4):

4 $$ {\boldsymbol{A}}_{\boldsymbol{\varphi}}^{\boldsymbol{S}}=\frac{A}{m}\varphi =\frac{A}{\rho \cdotp V}\varphi =\frac{4{r}^2\pi }{\rho \cdotp \frac{4{r}^3\pi }{3}}\varphi =\frac{3}{\rho \cdotp r}\varphi =\varphi \frac{6}{\rho \cdotp d}\left[\frac{m^2}{kg}\right]=\boldsymbol{\varphi} \frac{\mathbf{6000}}{\boldsymbol{d}\boldsymbol{\rho }}\left[\frac{m^2}{g}\right] $$

where *A* is the surface area and *V* is the volume of a particle, *2r = d* is the particle diameter [nm], *ρ* is the particle density (g/cm^3^, *ρ*_*SiO2*_ *= 2.32 g/cm*^*3*^) and φ = 0.9069 (reducing the area with the glass beads’ contact points in 3D), assuming hexagonal and monoparticular adhesion of spherical particles (see ESM Fig. S5a and b). Similarly, the cross-sectional surface area of a particle with a diameter of *d [nm]* and density of *ρ (g/cm*^*3*^*, ρ*_*Au*_ *= 19.3 g/cm*^*3*^) can be determined by eq. (5) (ESM Fig. S5c):

5 $$ {\boldsymbol{a}}_{\boldsymbol{NP}}^{\boldsymbol{s}}=\frac{A_p}{m}=\frac{r^2\pi }{\rho \cdotp \frac{4{r}^3\pi }{3}}=\frac{3}{\rho \cdotp 4\cdotp r}=\frac{3}{\rho \cdotp 2\cdotp d}\left[\frac{m^2}{kg}\right]=\frac{\mathbf{3000}}{\mathbf{2}\boldsymbol{d}\boldsymbol{\rho}}\left[\frac{m^2}{g}\right] $$

where *A*_*p*_ is the projection area of a sphere. The (theoretically calculated) maximal monoparticular (Θ = 1) adhered amount of nanoparticles can be determined by the ratio of these two values, as described by eq. (6). The results are presented in Table 1.

6 $$ {m}_{mp, th}^{NP}=\frac{A_{\varphi}^s}{a_{NP}^s} $$

**Table 1:** The cross-sectional surface area of the AuNPs (a^s^_NP_), GB diameter (d_GB_), specific surface area of GB (A^S^) and the maximal adhered mass of AuNP (m^NP^_mp,th_) in the case of the different plasmonic NPs with different diameters (d_NP_)

Sol	d_NP_ [nm]	a^s^_NP_ [m^2^/g]	d_GB_ [μm]	A^S^ [m^2^/g]	m^NP^_mp,th_ [mg/g]
AuNP(−)	14 ± 1	5.6 ± 0.4	55.7 ± 9.2	0.042 ± 0.008	7.6 ± 1.9
			273.7 ± 37.6	0.009 ± 0.001	1.5 ± 0.3
AuNP(+)	51 ± 13	1.5 ± 0.4	55.7 ± 9.2	0.042 ± 0.008	27.6 ± 12.9
			273.7 ± 37.6	0.009 ± 0.001	5.6 ± 2.4

### Adhesion in equilibrium

The adhesion of the gold nanoparticles on the surface of GB274(+) glass beads was investigated in equilibrium conditions (in a non-flow system), as described in [45], at different pH values.

Before measurement the GB274(+) samples were placed into an oven at 373 K for 4 h. Measured amounts (m = 0.5 ± 0.009 mg) of heat-treated GB274(+) sample was placed into vessels followed by introduction of the pre-prepared AuNP(−) gold sols (V = 10 mL) with known concentrations (*N* = 5, c_0_ = 20, 40, 50, 60 and 80 mg/L; three series were investigated at different pH values: pH = 3, 5 and 7). The vessel was closed and equilibrated for 5 h with slight magnetic stirring, while the equilibrium pH value was checked and corrected to the initial value in every hour. The change in the concentration of the gold sol due to adsorption was determined by UV-Vis photometry, measuring the extinction values of the supernatant (c_e_). The amount corresponding to the decrease in concentration (c_0_-c_e_) is equal to the specific adhered mass of AuNP(−)s (m^NP^_exp_, mg/g).

7 $$ {m}_{exp}^{NP}=\frac{\left({c}_0-{c}_e\right)\cdotp V}{m_{GB}} $$

The surface coverage can be calculated as the ratio of this value and the theoretically calculated maximally adhered amount: *Θ = m*^*NP*^_*exp*_*/ m*^*NP*^_*mp,th*_. Table 2 shows the systematic increase of the adhered amount (and the surface coverage) due to the pH decrease, which result was verified by the flow system measurements.

**Table 2:** Specific adhered mass of gold nanoparticles (m^NP^_exp_) and surface coverage (Θ) values in the case of GB274(+) glass beads and *d* = 14 nm AuNP(−) gold nanoparticles, at different pH values, in equilibrium conditions

GB274+/AuNP−	m_GB_(g)	pH	m^NP^_exp_ (mg/g)	θ
+/−	0.5000	7	0.18	0.12 ± 0.03
+/−	0.5086	5	0.43	0.29 ± 0.06
+/−	0.5050	3	0.72	0.48 ± 0.11

### Adhesion in a flow system

#### Charge dependency

**Fig. 3:**
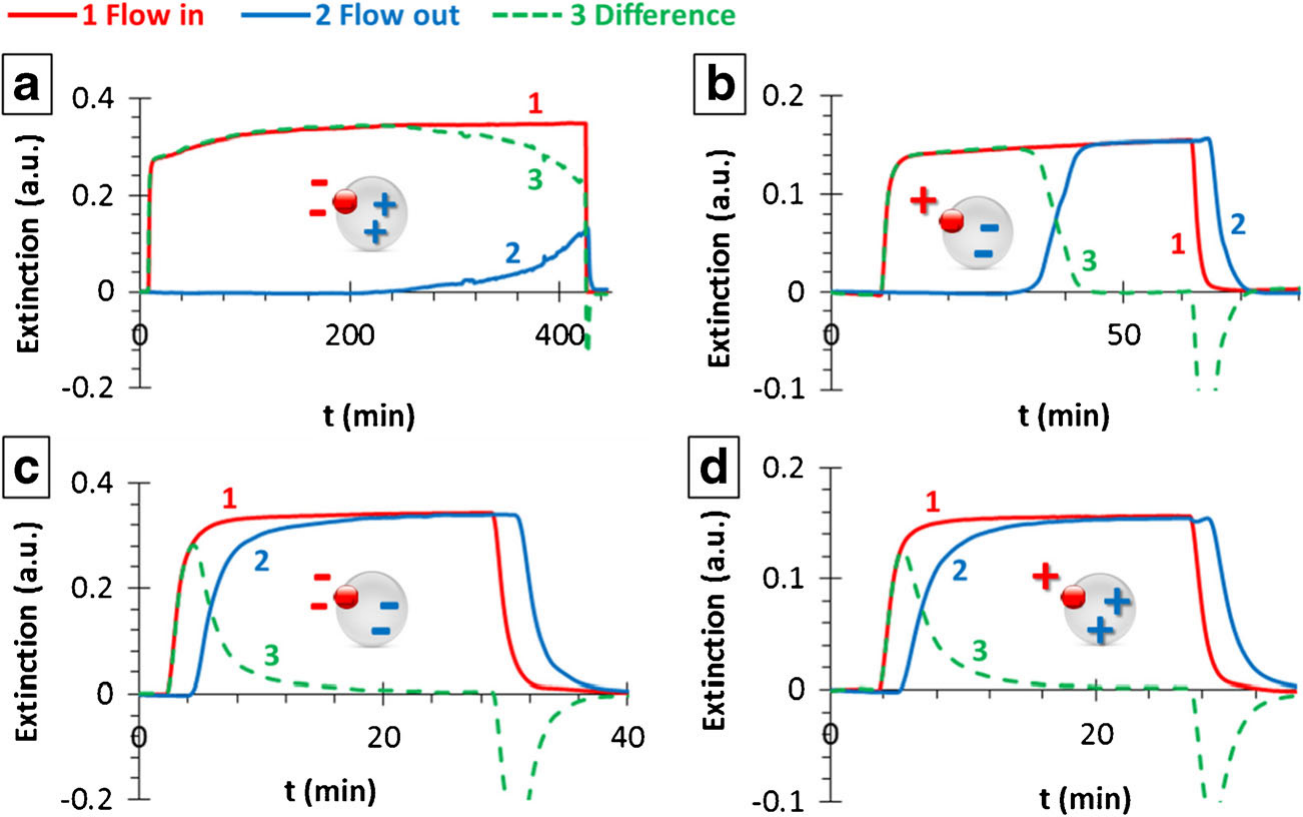
Results of the charge dependency of the gold nanoparticle adhesion experiments for AuNP(−)/GB56(+) (**a**), AuNP(+)/GB56(−) (**b**), AuNP(−)/GB56(−) (**c**) and AuNP(+)/GB56(+) (**d**) pairs (AuNP: nanoparticle, GB: stationary phase): flow-in (red), flow-out (blue) and difference curves (green)

Figure 3 shows the results of the surface charge dependency experiments in the case of AuNP/GB56: −/+, ±, −/− and +/+ pairs. As expected, both the time and the extent of the adhesion are negligible in the case of the uniformly charged units: the adhered mass of AuNPs is in the few μg/g range, while the adhesion time is less than 15 min. Slightly stronger interaction was observed for the AuNP(+)/GB56(−) pair, and a significant adhesion interaction was found between the AuNP(−)s and GB56(+)s. The results are summarized in Table 3. Having regard to the extremely high adhered amount of AuNP(−) particles due to the strong attraction between the negatively charged AuNPs and the PEI modified, therefore positively charged GBs even at pH = 7 value, and assuming that decreasing the pH will cause a significant increase in the adhered amount of gold nanoparticles, the pH dependency experiments were carried out by using the GB274(+) glass beads, because they have lower surface energy and specific surface charge.

**Table 3:** Summarizing table of the data obtained from the experiments carried out by using GB56 glass beads and pH = 7 AuNP sols with different surface charge combinations: m_AuNP_ is the calculated adhered mass of AuNPs, m_GB_ and ε are the mass and the porosity of the stationary phase, respectively, m^NP^_exp_ is the calculated specific adhered mass of AuNPs and t_adh_ is the adhesion process time

AuNP	Glass beads	m_AuNP_ (mg)	m_GB_ (g)	ε (%)	m^NP^_exp_ (mg/g)*	t_adh_ (min)
AuNP(−)	GB56(+)	1.935	4.61	39.3	> 0.419	> 400
AuNP(+)	GB56(−)	0.229	4.58	39.7	0.050	36
AuNP(−)	GB56(−)	0.035	4.54	40.3	0.008	14
AuNP(+)	GB56(+)	0.013	4.44	41.6	0.003	14

*Inaccuracy 10 μg*/g*

#### pH dependency

**Fig. 4:**
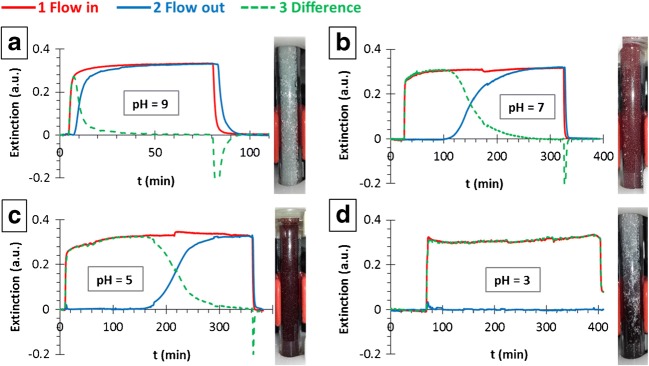
Results of the pH dependency of the gold nanoparticle adhesion experiments for AuNP(−)/GB274(+) pairs in the case of pH = 9 (**a**), 7 (**b**), 5 (**c**), and 3 (**d**) (AuNP: nanoparticle, GB: stationary phase): flow-in (red), flow-out (blue) and difference curves (green)

For the investigation of the pH dependency of the adhesion process gold sols of 20 mg/ml concentration were used, at different pH levels (pH = 3, 5, 7, and 9) on the larger (GB274) glass beads. A rapid increase in the adhesion time was observed with the decreasing pH, while with increasing the pH the amount of adhered gold greatly decreased, which can be explained by the pendant -NH_2_ groups of the PEI, which covers the surface of the glass beads becoming increasingly protonated with the decreasing pH, while the –COOH and –COO^**−**^ groups of the citrates that covers the gold nanoparticles are neutrally or slightly negatively charged (*pK*_*A*_ *= 2*) [21], thus allowing the formation of a strong interaction. Obviously, the increasing pH causes a deprotonation on the –NH_2_ groups of the PEI, the net positive charges are compensated by the negative surface charges of the glass beads, thus the adhesion interaction weakens. There is also a visually detectable color change of the adhered gold at different pH levels (Fig. 4): at pH *=* 9 the color barely changes, at pH *=* 7 and 5 the specific wine red color of the citric gold nanoparticles could be observed, with a deeper color at the lower pH, which indicated the larger amount of adhered gold, while at pH *=* 3 the color became dark purple, which can be an evidence of the strong aggregation of the particles on the surface of the glass beads (the degree of gold nanoparticle aggregation both in the sol and on the surface can be checked by the color, i.e. by the shape of the extinction spectra. During the measurements no change was observed, neither in the flow-in nor in the flow-out branch, indicating an unchanged aggregation state and nanoparticle size, except in the case of pH = 3, but only for the adhered NPs, not in the sol). The state of the column shows that this process did not saturated even after more than 300 min. The results are summarized and compared with the data obtained from the experiments carried out in the equilibrium conditions in Table 4. The result of the static and dynamic investigations are in a good agreement, the slight differences in the surface coverage values can be explained by the inaccuracy of the measurement.

**Table 4:** Summarizing table of the data obtained from the experiments carried out by using GB274(+) glass beads and pH = 3, 5, 7 and 9 AuNP(−) sols: ε is the porosity of the stationary phase, m_AuNP(−)_ is the calculated adhered mass and m^NP^_exp_ is the calculated specific adhered mass of AuNPs, t_adh_ is the adhesion process time, color shows the color of the stationary phase after the adhesion process, Θ_flow_ and Θ_eq_ show the calculated surface coverage values obtained by the flow system and equilibrium experiments

pH	m_AuNP(−)_ (mg)	m_GB274(+)_ (g)	ε (%)	m^NP^_exp_ (mg/g)*	t_adh_ (min)	color	θ_flow_	θ_eq_**
3	> 3.050	4.69	37.8	> 0.65	> 350	purple	> 0.43 ± 0.09	0.48 ± 0.11
5	2.013	4.76	36.9	0.407	150	wine red	0.27 ± 0.08	0.29 ± 0.06
7	1.159	4.73	37.3	0.245	80	red	0.16 ± 0.04	0.12 ± 0.03
9	0.061	4.67	38.1	0.013	5	light red	–	–

*Inaccuracy 10 μg/g, **data fr*om* Table 2

#### Reflectometric interference spectroscopy measurements

**Fig. 5:**
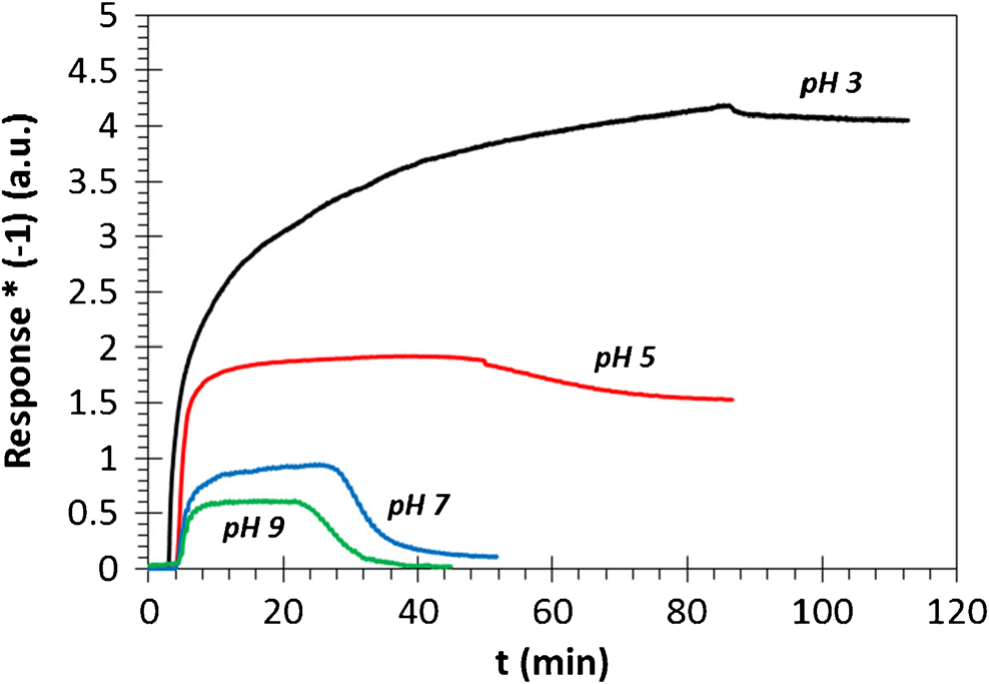
Results of the nanoparticle adhesion measurements on the surface of d = 450 nm in diameter SiO_2_ NPs: polarization reflectometric interference spectroscopy sensorgrams for pH = 9 (green), 7 (blue), 5 (red) and 3 (black) AuNP(−) sols

The experiments in the aqueous flow system (bulk, 3D) were verified by polarization reflectometric interference spectroscopy (PRIfS) measurements, with the difference that the surface for the adhesion was not GB, but a thin film formed by d = 450 nm SiO_2_ particles. After the preparation of the thin film the surface was modified by PEI by dip-coating technique [31]. Given the fact that this method is based on thin film technique, both the flow rate and the concentration of the AuNP(−) sol were reduced compared with the previously presented flow-system. The flow rate in these experiments was *I* = 50 μL/min and the concentration of the gold sol was 2 mg/L. Figure 5 shows the results: similarly to the results presented in Ch. “pH dependency”, significantly increasing adhered amounts of AuNP(−)s and adhesion process times were observed with the decrease in pH, as expected. The explanation is the same: the decreasing pH causes a protonation on the –NH_2_ groups of the PEI; thus, the adhesion interaction between the protonated –NH_3_^+^ groups and the AuNP(−)s intensifies.

## Conclusions

In this work a model experiment of adhesion of gold nanoparticles on glass bead solid surfaces was presented, addressing the effect of the charge state of the components (±, −/+, −/−, +/+), the glass bead size (d = 56 and 274 μm) and the pH (= 3, 5, 7, and 9) on the adhesion process in both (static) equilibrium and (dynamic) flow systems. The dynamic measurements were carried out in a liquid-flow platform, which consists of a column containing glass beads as a solid phase, with a concentration detection on both ends, which allows the investigation of the adhered amount of gold nanoparticles and the strength of the adhesion. The size of the glass beads seemed to have a significant effect on the amount of adhered gold: with the increasing size, the adhered amount decreased, due to the smaller surface energy and charge density of the larger glass beads. The investigation of the effect of charge served the expected results: the interaction between the equally charged component was negligible, while the interaction of the opposing charges was significantly stronger, especially in case of the AuNP(−) and the PEI-modified glass beads, due to the protonatable groups of the polymer. A similar effect was observed in the case of the pH dependency investigation, where the decrease of the pH resulted in a rapid increase of the protonation of the polyelectrolyte, as well as the adhered amount of gold and the level of aggregation. The result of the static and dynamic investigations were in good agreement, despite the non-equilibrial nature of the dynamic system; therefore, the adhesion of these components seems to be irreversible. The slight differences in the surface coverage values can be explained by the inaccuracy of the measurement. During the static measurements, a precipitation reaction occurred in the case of the gold sol produced by a metal-matrix reduction reaction, which prohibited the measurement of adhesion; however, in the case of the gold sol reduced and stabilized by citrate and the PEI-modified glass beads, the adhesion was easily observable and the outcome showed good agreement with the results of the dynamic measurements. In this case, the precipitant formation was restrained by the brief interaction of the nanoparticles and the glass beads, which prevented the occurrence of the surface reaction. The results of the 3D measurements were verified by using reflectometric interference technique: both the adhered amount of AuNP(−)s and the adhesion process time were significantly increased with the decrease in pH of the gold sol, because the decreasing pH causes a protonation on the –NH_2_ groups of the PEI, thus the adhesion interaction between the –NH_3_^+^ groups and the AuNP(−)s intensifies.

## Electronic supplementary material

ESM 1 (PDF 858 kb)
