# Supplementary material for: Fast optical method for characterizing plasmonic nanoparticle adhesion on functionalized surfaces
Source: Anal Bioanal Chem. 2019 Dec 24;412(14):3395–404. doi: 10.1007/s00216-019-02307-x (PMC7214493; doi:10.1007/s00216-019-02307-x)
Supplement: Supplementary file 1 — (PDF 858 kb) [file 216_2019_2307_MOESM1_ESM.pdf]

Analytical and Bioanalytical Chemistry

Electronic Supplementary Material

Fast optical method for characterizing plasmonic nanoparticle adhesion on functionalized surfaces

László Mérai, László Janovák, Dániel Sándor Kovács, Imre Szenti, Lívia Vásárhelyi,
Ákos Kukovecz, Imre Dékány, Zoltán Kónya, Dániel Sebők

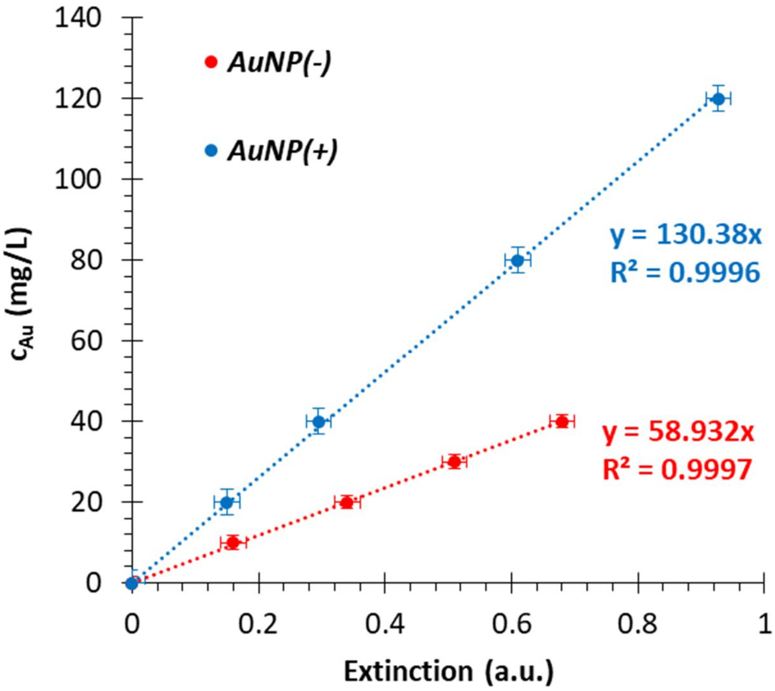

Fig. S1 Concentration (c, mg/L) vs. extinction calibration curves for the AuNP(-) (blue) and AuNP(+) (red) gold nanoparticle sols

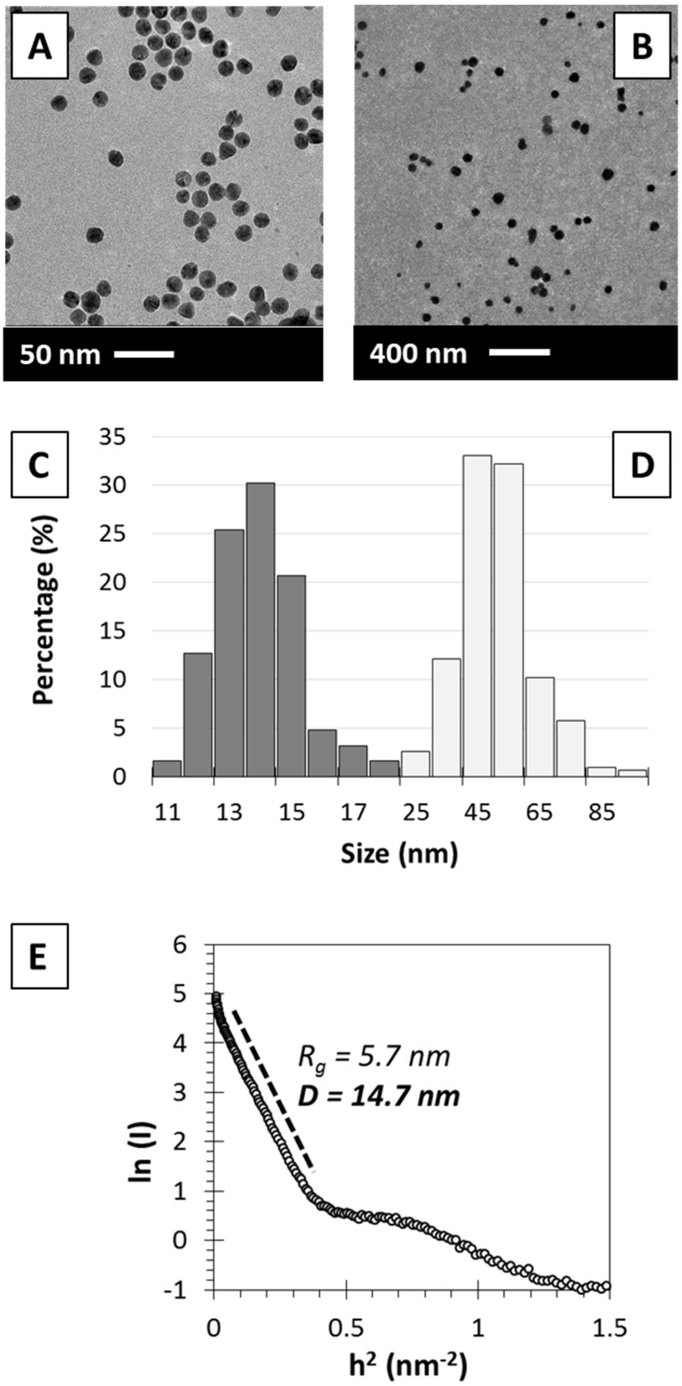

Fig. S2 Characterization of the gold nanoparticles: Representative TEM images of AuNP(-) (A) and AuNP(+) (B) gold nanoparticles and the size distributions of AuNP(-)s (C) and AuNP(+)s (D) determined from TEM images. The Guinier plot of the SAXS curve of the dried AuNP(-) powder sample to determine the average particle diameter (E)

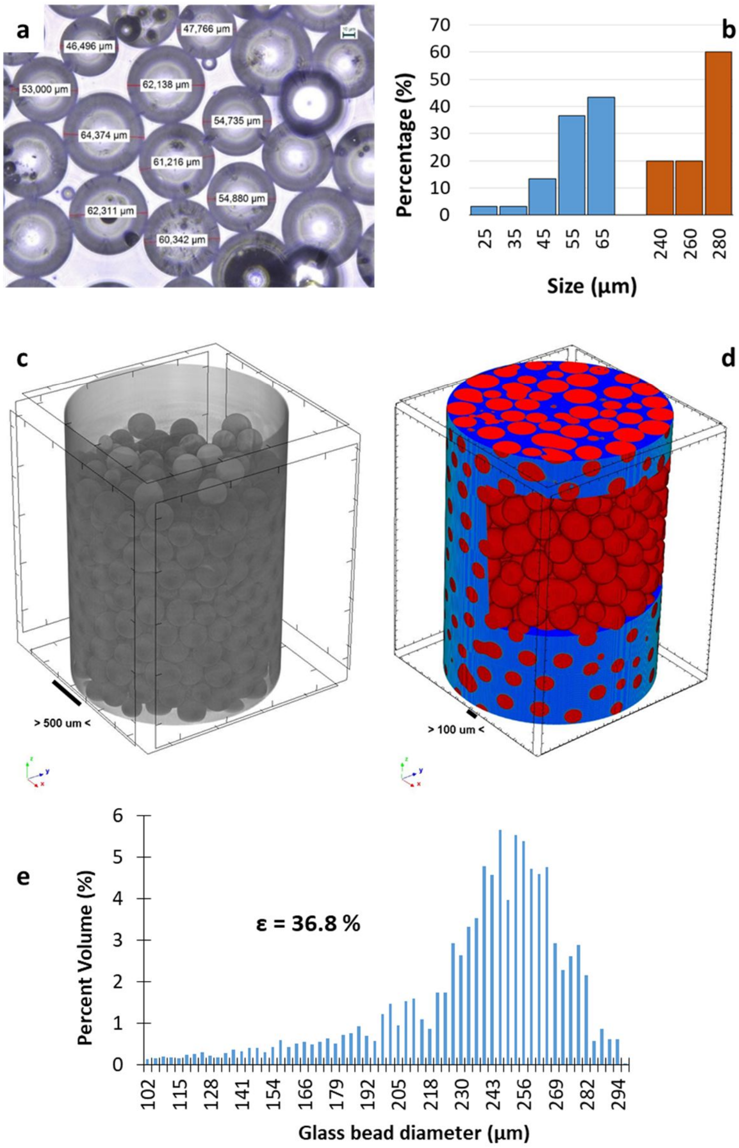

Fig. S3 Characterization of the glass beads: Representative optical microscopy image of the GB56 glass beads (a), the size distribution of the GB56 (blue) and GB274 (orange) glass beads determined from the microscope images. The 3D volume rendered image of the GB274 glass beads in a d = 2 mm in diameter glass capillary (c) and the volume of interest (VOI) (d, red: GB, blue: void) to determine the size distribution and the porosity of the stationary phase based on micro-CT measurements. The size distribution of GB274 glass beads and the porosity (ε) of the loading determined from micro-CT measurements (e)

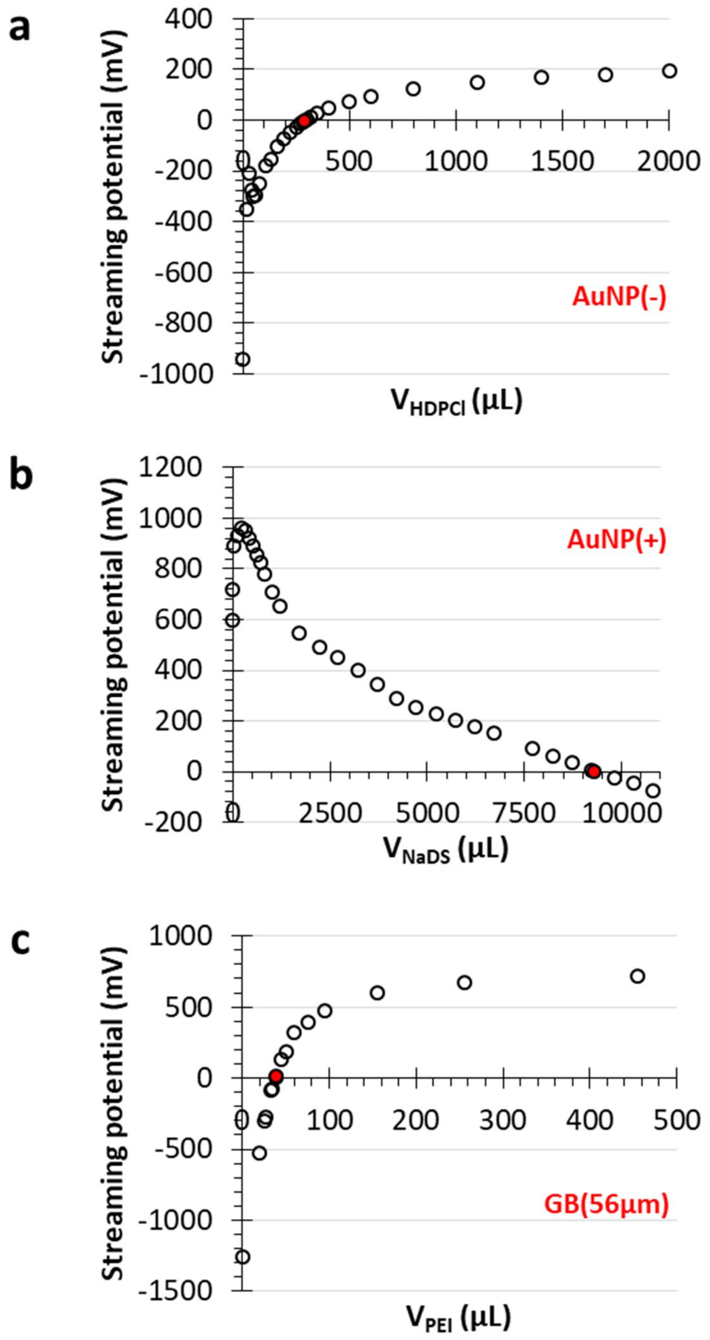

Fig. S4 Characteristic surface charge titration curves of AuNP(-) (a), AuNP(+) (b) and GB56 glass beads (c) : measured streaming potential values as a function of the added titrant volumes. The surface charge neutralization is indicated by the red data points. The titrants were 0.01 % HDPCl solution, 0.01 % NaDS solution and 0.01 % PEI solutions, respectively

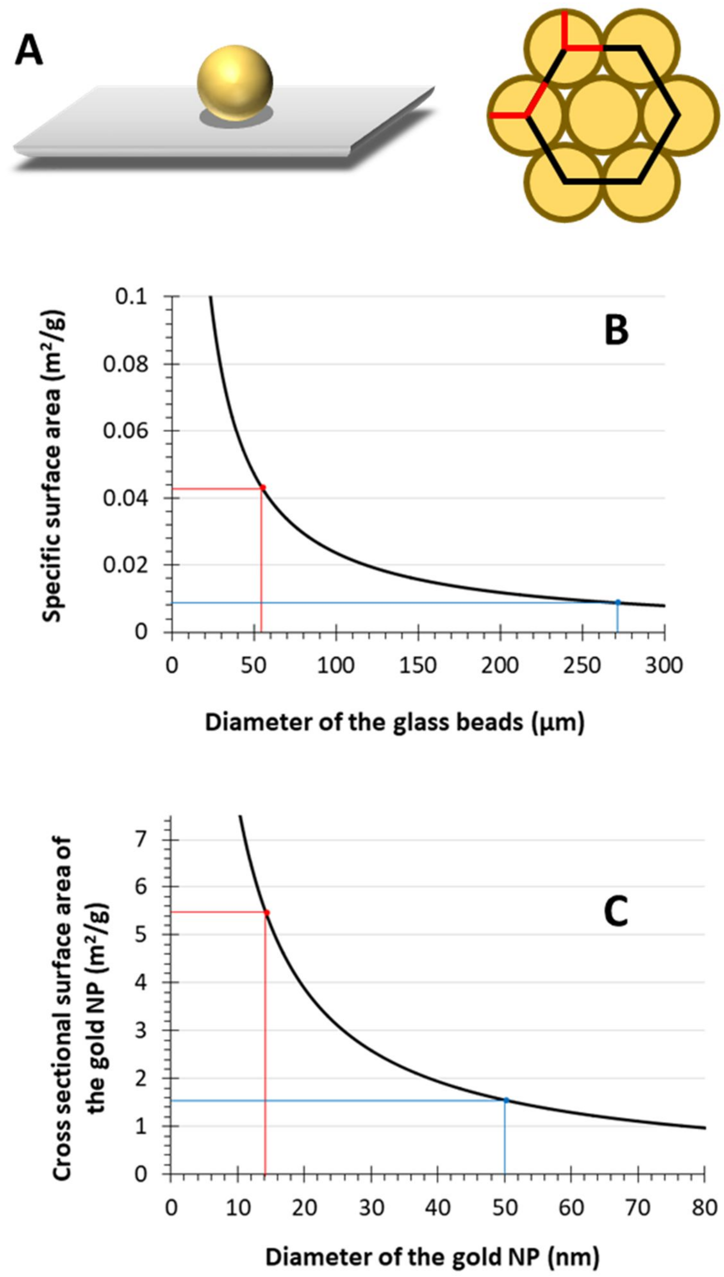

Fig. S5 Schematic view of the hexagonal arrangement of the gold nanoparticles on the surface of the glass beads (A). The specific surface area of the glass beads (B) and the cross-sectional surface area of the gold nanoparticles (C) as the function of the diameter

B: red and blue lines indicate the data for GB56 and GB274, respectively.

C: red and blue lines indicate the data for AuNP(-) and AuNP(+), respectively.
